# Supplementary material for: Phase I/II Study of LDE225 in Combination with Gemcitabine and Nab-Paclitaxel in Patients with Metastatic Pancreatic Cancer
Source: Cancers (Basel). 2021 Sep 28;13(19):4869. doi: 10.3390/cancers13194869 (PMC8507646; doi:10.3390/cancers13194869)
Supplement: Supplementary file 1 [file cancers-13-04869-s001.zip › cancers-1395136-SI-done.pdf]

cancers

MDPI

Article

Phase I/II Study of LDE225 in Combination with Gemcitabine and Nab-Paclitaxel in Patients with Metastatic Pancreatic Cancer

Esther N. Pijnappel [1], Nienke P.M. Wassenaar [2], Oliver J. Gurney-Champion [2], Remy Klaassen [1], Koen van der Lee [1], Marjolein C.H. Pleunis-van Empel [3], Dick J. Richel [1], Marie C. Legdeur [3], Aart J. Nederveen [2], Hanneke W.M. van Laarhoven [1] and Johanna W. Wilmink [1,*]

Supplementary Materials:

The following are available online at www.mdpi.com/xxx/s1, Table S1: Summary of the relevant MRI parameters for IVIM-DWI, DCE MRI and T1 mapping; Table S2. CA 19.9 levels at baseline and at evaluation of the 36 patients who received a baseline MRI scan.

Table S1. Summary of the relevant MRI parameters for IVIM-DWI, DCE MRI and T1 mapping.

	IVIM-DWI	DCE	T1 look-locker
FOV (RL × AP) (mm²)	432 x 108	400 x 400	400 x 350
Acquisition matrix	144 x 34	160 x 160	132 x 116
Slices	18	30	13
Slice thickness/gap (mm)	3.7/0.3	2.5 (5.0 non-interpolated)	5.7 (11.4 non-interpolated)
TR/TE (ms)	>2200/45	3.19/2.0	3.5/1.6
FA (°)	90	20	8
Parallel imaging	1.3 (AP)	3.6/1.5 (RL/AP)	3/1.3 (RL/AP)
Respiratory compensation	Respiratory trigger (navigator)	Postprocessing	1 breath-hold
Fat saturation	Gradient reversal during slice selection + SPIR	-	-
b-values (s/mm²) and directions/averages	0 (15), 10 (9), 20 (9), 30 (9), 40 (9), 50 (9), 75 (4), 100 (12), 150 (4), 250 (4), 400 (4), 600 (16)	-	-
Diffusion times δ/Δ (ms)	10.1/22.6	-	-

FOV: field of view, RL: right left, AP: anterior posterior, TR: repetition time, TE: echo time, FA: flip angle, SPIR: spectral presaturation with inversion recovery.

Table S2. CA 19.9 levels at baseline and at evaluation of the 36 patients who received a baseline MRI scan.

Patient	Baseline CA 19-9 in kU/L	Evaluation (8 weeks) CA 19-9 in kU/L
1.	9	14
2.	1667	454
3.	1453	1256

4.	352	293
5.	171394	217
6.	N.D.	N.D.
7.	N.D.	N.D.
8.	171088	57801
9.	11260	5800
10.	57300	52246
11.	3380	13371
12.	745	786
13.	4744	719
14.	N.D.	N.D.
15.	N.D.	N.D.
16.	419660	N.D.
17.	N.D.	N.D.
18.	N.D.	N.D.
19.	6203	1681
20.	N.D.	N.D.
21.	25697	N.D.
22.	N.D.	1814
23.	591	262
24.	2252	1125
25.	9250	5977
26.	N.D.	N.D.
27.	1107	2321
28.	904	1076
29.	30	N.D.
30.	4088	182
31.	13522	208
32.	180	194
33.	8583	8876
34.	3	6
35.	4077	864
36.	101833	68696

N.D.: not defined.
